# Comparison of Children’s and Parents’ Perceptions of Appearance Related to Trunk Deformity in Patients with Idiopathic Scoliosis Using the TAPS Questionnaire: Retrospective Observations

**DOI:** 10.3390/jcm15218257

**Published:** 2026-10-23

**Authors:** Marianna Białek, Sylwia Piorun, Ewelina Białek-Kucharska, Małgorzata Poczynek, Justyna Pękala, Tomasz Kotwicki

**Affiliations:** 1 FITS Method, 59-411 Paszowice, Poland; vewelinav@o2.pl; 2 Aktis, 59-220 Legnica, Poland; spiorun@onet.pl; 3 FizMal, 59-300 Lubin, Poland; gosallegro@gmail.com; 4 Kinetik, 59-400 Jawor, Poland; biuro.kinetikjp@gmail.com; 5 Department of Spine Disorders and Pediatric Orthopedics, Poznań University of Medical Sciences, 61-701 Poznan, Poland; kotwicki@ump.edu.pl

**Keywords:** idiopathic scoliosis, functional individual therapy of scoliosis (FITS Method), physiotherapeutic scoliosis specific exercise (PSSE), trunk appearance perception scale (TAPS)

## Abstract

**Background/Objectives**: Idiopathic scoliosis affects musculoskeletal biomechanics and trunk appearance. Patient-reported outcome measures, including the Trunk Appearance Perception Scale (TAPS), are important for assessing health-related quality of life and monitoring treatment. This study compared children’s and parents’ perceptions of trunk appearance before and after 5-day scoliosis-specific exercises according to Functional Individual Therapy of Scoliosis (FITS Method). Additionally, changes in the perception of trunk appearance by patients and parents were evaluated in relation to brace use. **Methods**: Ninety children with idiopathic scoliosis (74 girls and 16 boys; mean age: 13.6 ± 2.1 years) and their parents participated. Sixty-two children used a Chêneau brace. Most participants (*n* = 71) had double-curve scoliosis; the mean thoracic and lumbar Cobb angles were 32.5° ± 12.9 and 32.2° ± 13.0, respectively. Children and parents completed the TAPS at the beginning and end of the program. **Results**: After the 5-day rehabilitation program, TAPS scores were significantly higher in both children and their parents than at baseline (*p* < 0.001). The change in scores from before to after therapy differed significantly between children and parents (*p* = 0.046). In the group of children treated with braces, the assessment of the impact was statistically significant for both the children and their parents (*p* < 0.001 in both cases). In the group of children not treated with braces, the assessment was statistically significant only for the parents (*p* = 0.003). **Conclusions**: 1. Following a 5-day rehabilitation stay, a beneficial change was observed in the perception of trunk appearance among children with idiopathic scoliosis and their parents. 2. Parents rated the change after the program slightly more positively than the children themselves. 3. The perception of trunk appearance among children treated with and without a brace was similar after the stay.

## 1. Introduction

### 1.1. Background

Idiopathic scoliosis is the most common spinal deformity in children and adolescents, accounting for approximately 80–90% of all scoliosis cases [1]. Although its aetiology remains incompletely understood, genetic, hormonal, neurological, and biomechanical factors are believed to contribute to its development [2]. Idiopathic scoliosis most commonly becomes apparent during periods of rapid growth, and its progression may lead to substantial trunk deformity, functional limitations, and reduced quality of life [3,4].

For many years, the assessment of scoliosis treatment outcomes was based primarily on radiological parameters, particularly the Cobb angle. More recently, increasing attention has been given to patients’ subjective perceptions of their own appearance and to the impact of the deformity on everyday functioning, both of which may influence quality of life [5,6]. Visible changes in body shape, such as asymmetry of the shoulders, scapulae, and waist, as well as the presence of a rib prominence, may contribute to body image disturbance, reduced self-esteem, difficulties in social relationships, and poorer psychological well-being [7,8]. This issue is particularly relevant during adolescence, when physical appearance plays an important role in identity formation and social acceptance [9].

In most cases, scoliosis does not cause health problems during growth, except in severe cases.

However, the resulting deformity often negatively affects adolescents’ body image and self-esteem, potentially leading to a reduced quality of life (QoL) and even mental health disorders [10].

Parents play an important role in the treatment of children and adolescents with idiopathic scoliosis. They are often the first to notice the deformity, participate in treatment-related decision-making, support the child during rehabilitation, monitor adherence to brace treatment recommendations, and observe changes in body appearance [11]. Therefore, parents’ perception of the deformity may influence both the course of treatment and the child’s overall functioning.

### 1.2. Objectives

1. To compare the subjective assessment of body posture by patients with idiopathic scoliosis and their parents, using the TAPS questionnaire before and after a 5-day scoliosis-specific therapy programme based on Functional Individual Therapy of Scoliosis (FITS).
2. Comparison of the agreement between child and parent assessments using the TAPS questionnaire.
3. To assess changes in the subjective perception of trunk appearance among patients and their parents following therapy, with separate analyses for patients treated with a brace and those not undergoing brace treatment.

## 2. Material and Methods

### 2.1. Study Design

A retrospective observational study was conducted to compare children’s and parents’ perceptions of trunk appearance before and after a 5-day rehabilitation program based on the Functional Individual Therapy of Scoliosis (FITS) Method, using the Trunk Appearance Perception Scale (TAPS).

### 2.2. Setting

The study protocol, including the retrospective analysis of medical records from rehabilitation programs conducted between 2022 and 2026, was approved by the Bioethical Commission at the University of Medical Sciences in Poznan (decision no. KB-549/26).

At the beginning and at the end of each rehabilitation programme, the children underwent clinical examinations performed by an experienced certified FITS therapist. In addition, both the children and their parents independently completed the TAPS questionnaire (without consulting each other) after receiving instructions from a physiotherapist.

### 2.3. Participants

The study included all patients aged 10–16 years with idiopathic scoliosis who participated in 5-day rehabilitation programmes based on the FITS method and had complete TAPS questionnaire data provided independently by both the patient and a parent or caregiver before and after therapy. Patients with scoliosis of an aetiology other than idiopathic, as well as those with incomplete clinical documentation, including missing TAPS questionnaire data, were excluded from the analysis.

Out of 105 patients attending the 5-day rehabilitation camps during the study period, 15 were excluded due to non-idiopathic scoliosis or structural spine disorders such as Scheuermann’s disease (*n* = 6), absence on the final day (*n* = 5), or incomplete/incorrect TAPS data (*n* = 4).

The study included 90 patients with idiopathic scoliosis and 90 of their parents or caregivers. The study group consisted of 74 girls (82.2%) and 16 boys (17.8%). The mean age was 13.6 years (SD = 2.1). Menarche had occurred in 33 girls (44.5%). Mean body weight was 48.7 kg, and mean height was 162.8 cm.

Among the participants, 71 had double-curve scoliosis. The mean thoracic Cobb angle was 32.5° (SD = 12.9), while the mean lumbar Cobb angle was 32.2° (SD = 13.0). Five patients had single-curve thoracic scoliosis, with a mean Cobb angle of 29.6° (SD = 16.5), whereas 14 patients had single-curve lumbar scoliosis, with a mean Cobb angle of 27.8° (SD = 7.1). The mean skeletal maturity assessed using the Risser sign was 2.2 (SD = 1.9). Sixty-two patients (68.9%) were treated with a brace. The mean angle of trunk rotation was 7.7° (SD = 4.4) in the thoracic region and 4.7° (SD = 3.9) in the lumbar region (Table 1).

Baseline TAPS scores were as follows: in the frontal plane, SET 1 (back view), the mean score was 3.37 (SD = 0.9) for children and 3.26 (SD = 0.9) for parents; in the transverse plane, SET 2 (forward-bending view), the mean score was 3.71 (SD = 0.7) for children and 3.48 (SD = 0.8) for parents; and in the frontal plane, SET 3 (front view), the mean score was 3.44 (SD = 0.8) for children and 3.36 (SD = 0.8) for parents (Table 2).

The study included 90 patients (*n* = 28 non-braced, *n* = 62 braced). Both groups had similar age (13.82 ± 2.48 vs. 13.50 ± 1.86 years) and Risser sign (2.11 ± 1.85 vs. 2.28 ± 1.98). Double-curve scoliosis was most common in both groups (64.3% non-braced, 85.5% braced). The braced group showed higher thoracic Cobb angles (34.55° ± 11.99° vs. 25.95° ± 13.94°) and thoracic ATR (8.34° ± 4.50° vs. 6.75° ± 4.50°). Lumbar Cobb angles were similar in groups (31.76° ± 11.36° vs. 30.88° ± 14.64°), while lumbar ATR was higher in the non-braced group (6.18° ± 4.64° vs. 4.89° ± 3.21°) (Table 3).

### 2.4. Interventions

The children participated in 5-day inpatient rehabilitation stay during which they followed a scoliosis-specific physiotherapy based on Functional Individual Therapy of Scoliosis (FITS Method).

The program included exercises aimed at increasing elongation, improving sensorimotor control and lower trunk stabilization, increasing trunk flexibility in the direction of scoliosis correction (shift and derotation of scoliosis), correcting the sagittal profile, practicing corrective movement patterns in functional positions, learning rotational breathing both with and without a brace, and developing active self-correction, defined as maintaining a corrected body posture during activities of daily living [12,13]. The children received 3 h of therapy per day. Parents or caregivers actively participated in all sessions and learned how to perform the exercises correctly with the child so that the program could subsequently be continued at home. Both the children and their parents also attended a lecture on scoliosis and the effects of therapy to improve their understanding of the condition and the treatment process.

At the beginning and end of each rehabilitation stay, patients underwent a clinical examination using the FITS method. The assessment included measurements of the distance from the plumb line to the gluteal cleft, the apex of the scoliosis curve, and the scapulae; assessment of scapular level; measurement of the angle of trunk rotation at the curve apex during the Adams test; and assessment of the sagittal plane using an inclinometer [12,13].

In modern physiotherapy and orthopaedics, increasing importance is being placed on standardized Patient-Reported Outcome Measures (PROMs) including the Trunk Appearance Perception Scale (TAPS), which is used in patients with scoliosis or other spinal deformities [6,14,15]. The TAPS allows patients to assess their own trunk deformity by comparing its appearance with illustrations representing different degrees of asymmetry.

**Figure 1:**
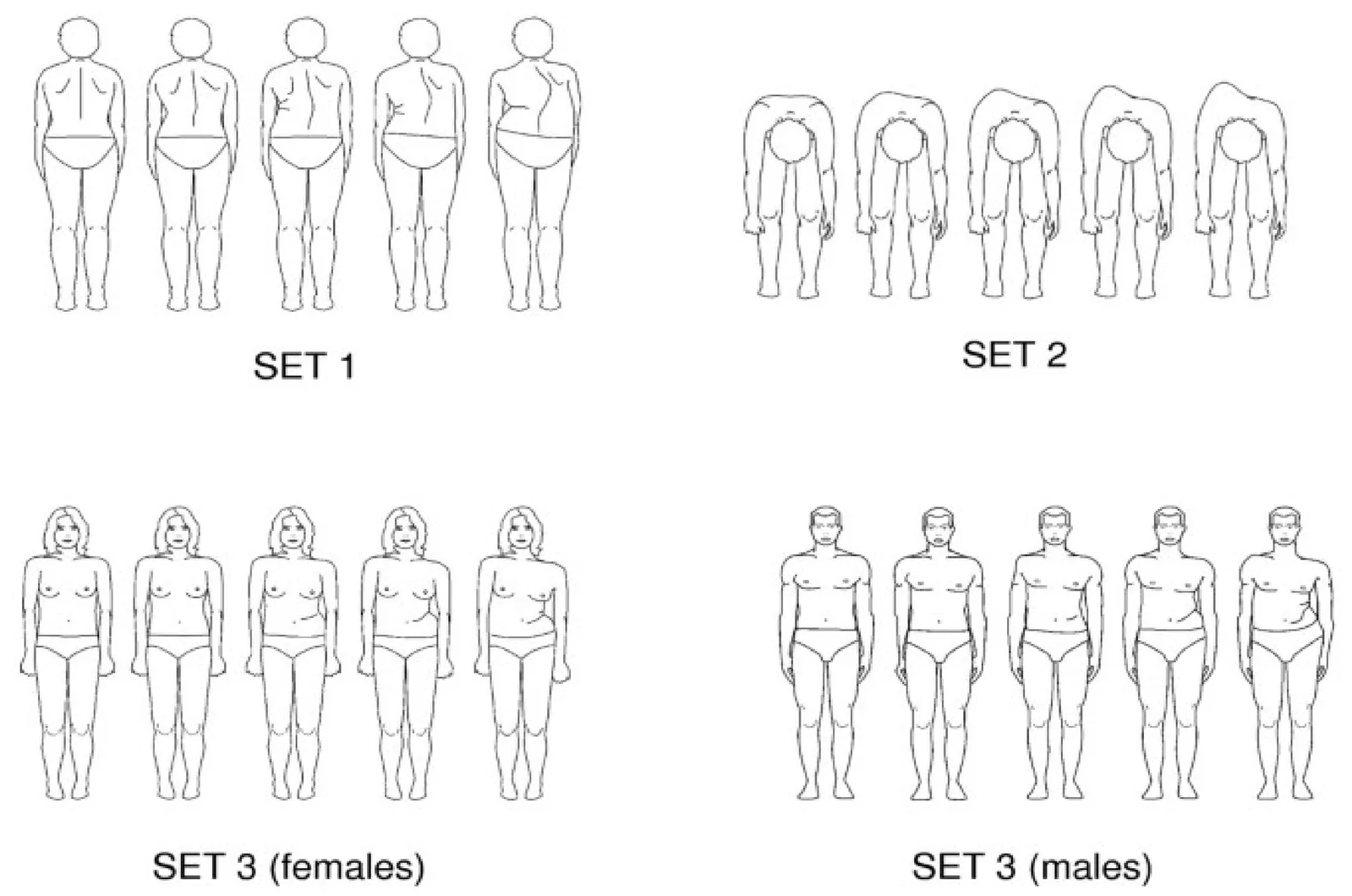
Trunk Appearance Perception Scale (TAPS).

The assessment procedure involves selecting the illustration that most closely corresponds to the actual deformity of the patient with scoliosis. The questionnaire consists of three sets of figures: SET 1 (back view in the frontal plane), SET 2 (view during forward bending in the transverse plane), and SET 3 (front view in the frontal plane) (Figure 1).

**Figure 2:**
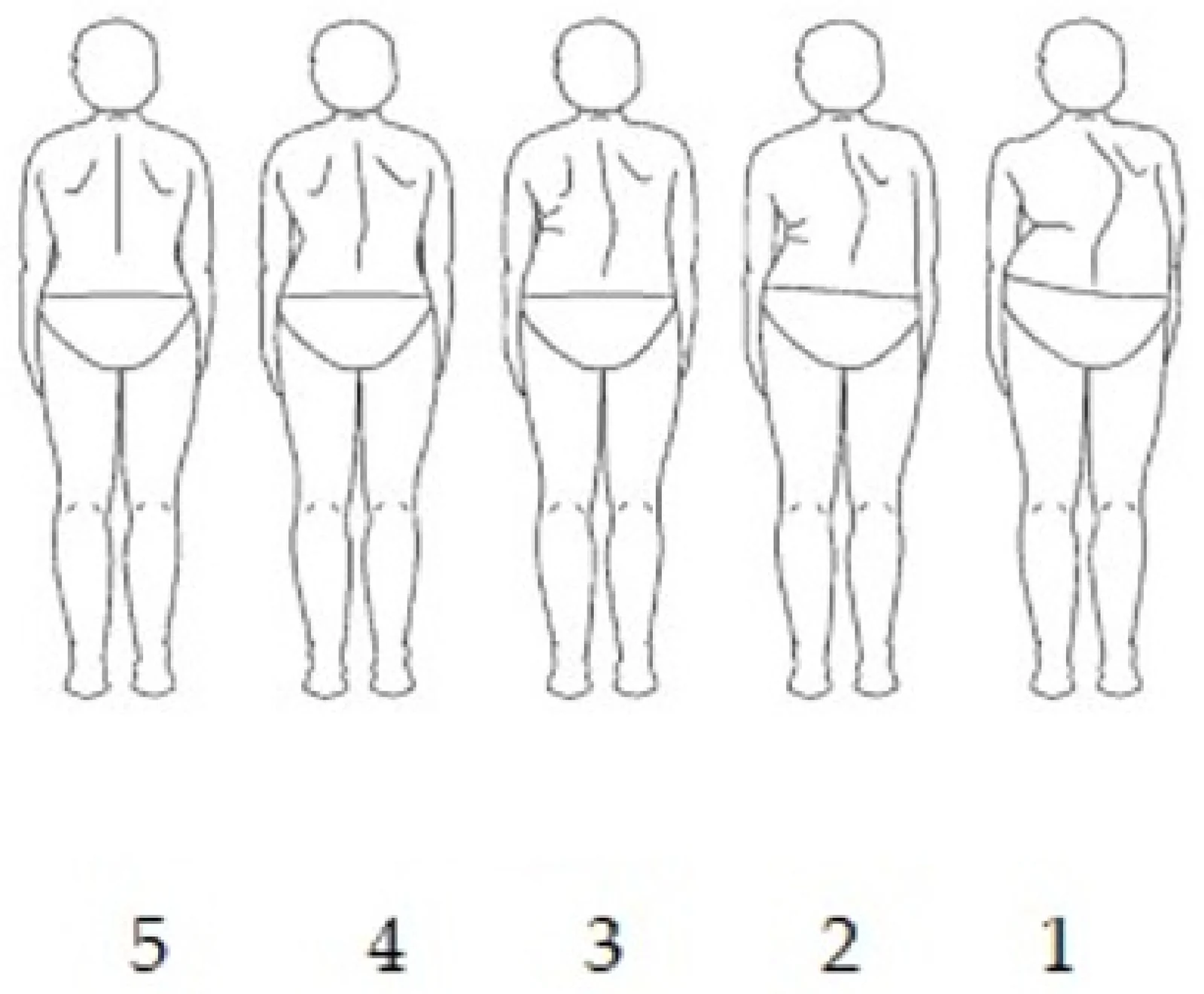
TAPS score range.

TAPS scores range from 1, representing the most severe perceived deformity, to 5, representing the least severe perceived deformity. A higher score indicates a more favourable perception of trunk appearance, whereas a lower score indicates a less favourable perception (Figure 2).

The TAPS questionnaire was completed twice—at the beginning and at the end of the rehabilitation program—by both the child and the same parent or guardian. Each individual completed a separate form without consulting the other and without seeing the results obtained on the scale. Both assessments were conducted prior to the clinical examination, at the start and end of the program, respectively.

Failure to answer any question resulted in the participant’s exclusion from the analysis; this applied to both children and parents or guardians. The study was retrospective in nature. Before the program began, parents or legal guardians signed a consent form for the assessments.

### 2.5. Statistical Methods

Statistical analysis of the collected data was performed using Statistica 13.3 software (StatSoft). Descriptive statistics are presented as mean values with standard deviations. The Wilcoxon signed-rank test, a non-parametric statistical test, was used for the analysis. This test was selected because the analysed variables were ordinal in nature. It was used to assess the statistical significance of changes in responses to the same item between two time points, namely before and after therapy, as well as to compare assessments of the same phenomenon made by two different individuals, i.e., the parent and the child.

Furthermore, the Mann–Whitney U test was used to compare results between independent groups—children with and without a brace. The level of statistical significance was set at *p* < 0.05.

## 3. Results

### 3.1. Outcome Data

TAPS SUM and TAPS SET 1, 2, 3 scores as well as the agreement between children’s and parents’ perceptions of trunk deformity, were compared before and after therapy.

### 3.2. Primary Results

**Figure 3:**
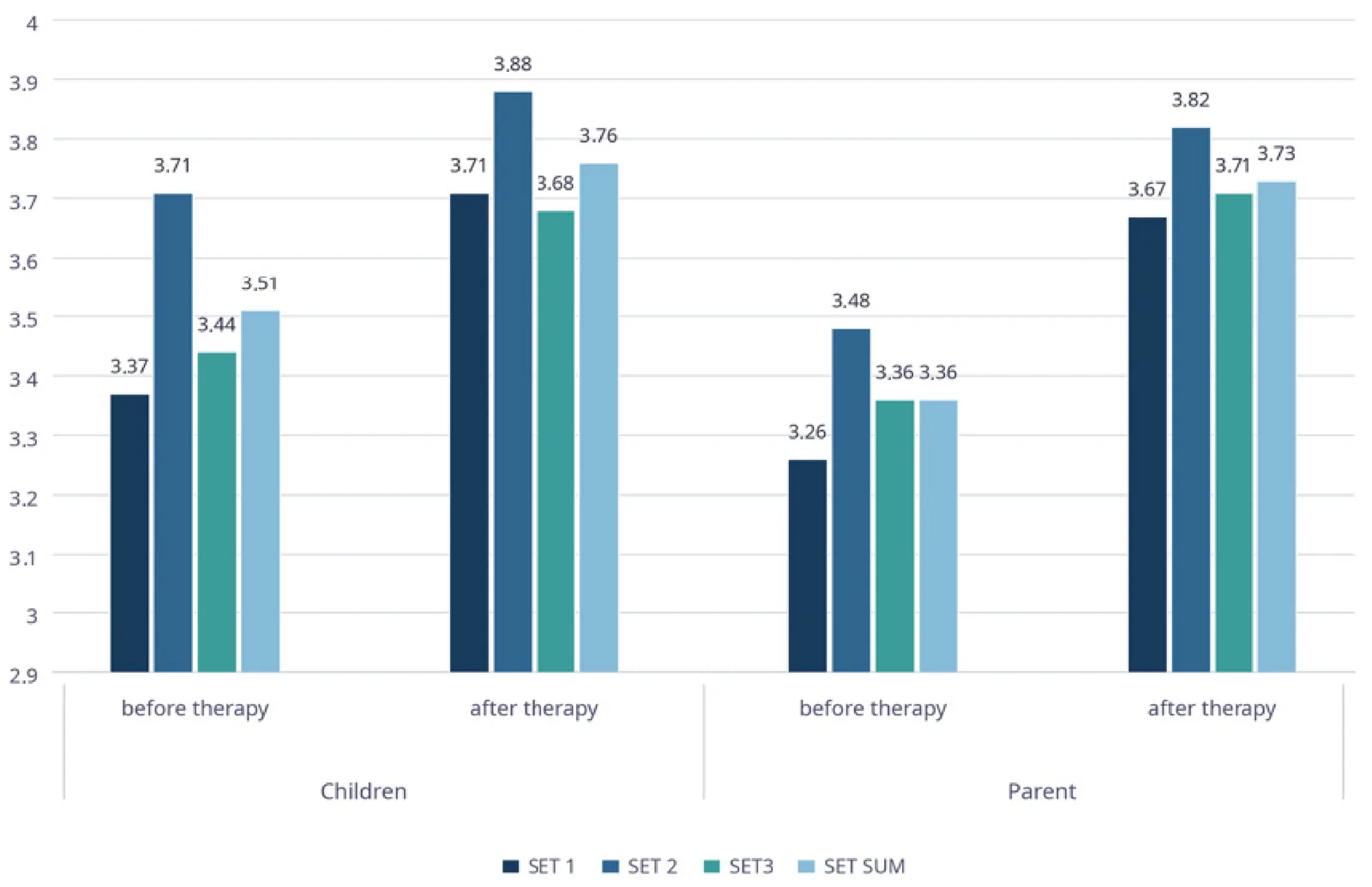
TAPS (SET 1, 2, 3) + TAPS SUM and the agreement of subjective trunk deformity assessment by children/parents pre- and post-therapy.

TAPS SUM (SET 1, 2, 3). The combined TAPS score for SET 1, SET 2, and SET 3 was analysed for both children and their parents at two consecutive measurements: before therapy, the mean overall score reported by the children was 3.51, increasing to 3.76 after therapy. The corresponding scores reported by the parents were 3.36 before therapy and 3.73 after therapy. A statistically significant difference between pre- and post-rehabilitation stay assessments was observed in both children and parents (*p* < 0.001), with higher overall TAPS scores recorded after therapy (Table 4, Figure 3). The change in scores from before to after therapy differed significantly between children and parents (*p* = 0.046) (Table 5). The absence of a minimum threshold for a clinically significant difference allows one to assume that any improvement in the assessment of clinical appearance can reinforce the motivation to continue treatment.

### 3.3. Secondary Results

TAPS–SET 1 (back view). For the frontal plane, the mean scores reported by the children were 3.37 before therapy and 3.71 after therapy, while the corresponding scores reported by the parents were 3.26 and 3.67, respectively. A statistically significant difference was observed between pre- and post-stay assessments in both children and parents (*p* < 0.001), with higher scores recorded after therapy.

TAPS–SET 2 (forward-bending view). For the transverse plane, the mean scores reported by the children were 3.71 before therapy and 3.88 after therapy. The corresponding scores reported by the parents were 3.48 and 3.82, respectively. A statistically significant difference between pre- and post-rehabilitation stay assessments was observed in both children (*p* = 0.041) and parents (*p* = 0.001). In both groups, higher scores were recorded after stay, indicating a more favourable perception of trunk appearance.

Parents assessed the rib hump more critically than the children both before and after camp. However, after rehabilitation stay, the assessments made by children and parents were more similar (Table 4, Figure 3).

TAPS–SET 3 (front view). For the frontal plane, the mean scores reported by the children increased from 3.44 before to 3.68 after stay. The corresponding scores reported by the parents increased from 3.36 to 3.71. A statistically significant difference between pre- and post- assessments was observed in both children (*p* = 0.005) and parents (*p* < 0.001). In both groups, higher scores were recorded after stay (Table 4, Figure 3).

The ratings given by the children and their parents did not differ significantly either before therapy (*p* = 0.094) or after its completion (*p* = 0.669). The change in the rating of trunk appearance following rehabilitation was greater according to the parents than in the children’s self-assessments (0.37 and 0.25 respectively; *p* = 0.046). This result indicates that parents perceived a greater improvement in their child’s trunk appearance than the children themselves did (Table 5).

Analysis of TAPS Results stratified by the group of children with and without a brace.

In the analysis of TAPS scores stratified by bracing status (braced vs. non-braced), differences in score changes were observed before and after the 5-day rehabilitation stay.

**Figure 4:**
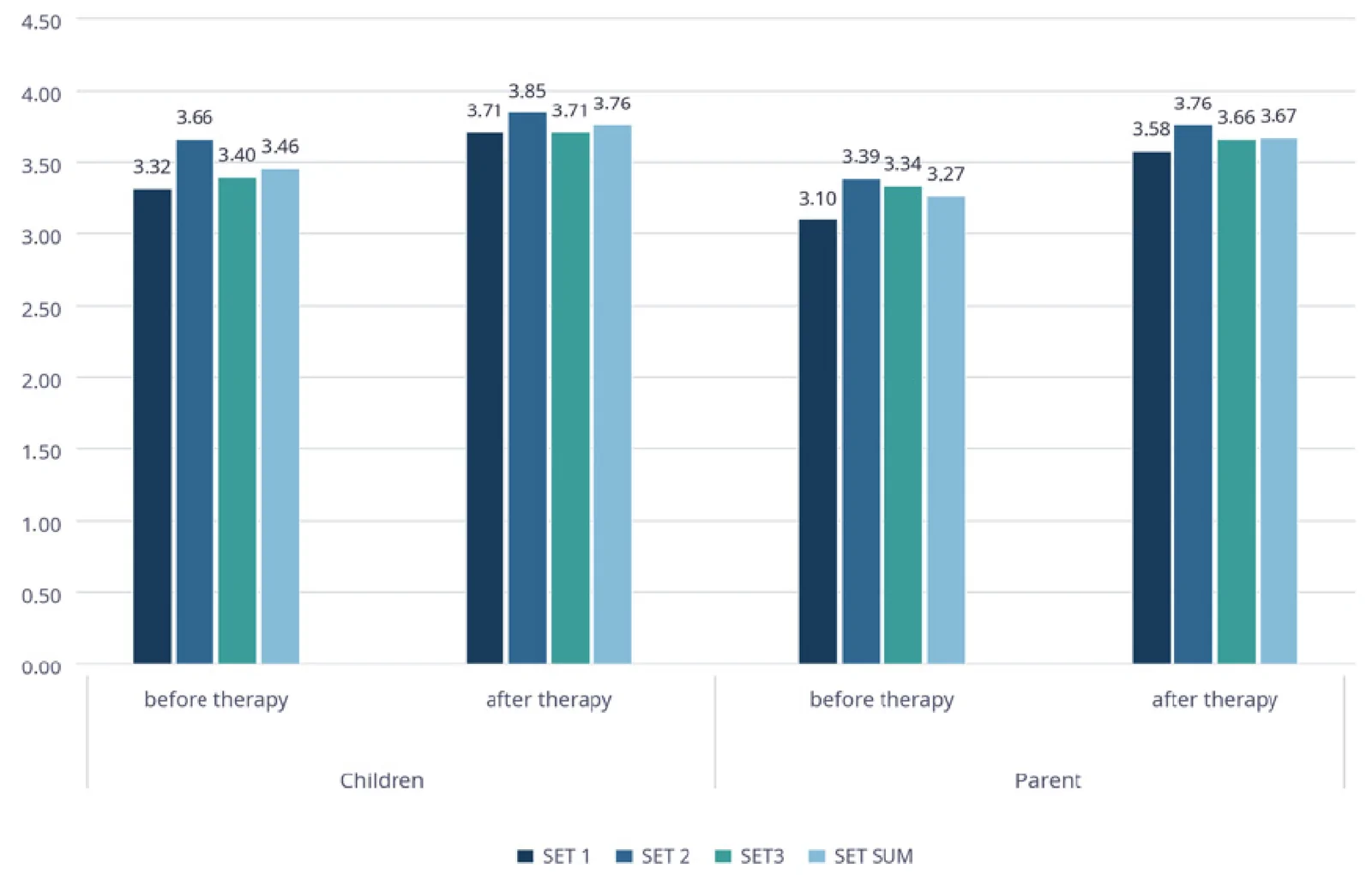
TAPS (SET 1, 2, 3) + TAPS SUM of braced children evaluated by children/parents before and after therapy.

In the braced group, mean TAPS scores increased across all three evaluated sets. In SET 1, children’s scores increased from 3.32±0.90 to 3.71±0.78 (p=0.001), and parents’ scores increased from 3.10±0.94 to 3.58±0.82 (p<0.001). In SET 2, the increases were from 3.66±0.65 to 3.85±0.72 (p=0.039) in children and from 3.39±0.80 to 3.76±0.76 in parents (p=0.003). In SET 3, scores also increased: in children from 3.40±0.82 to 3.71±0.80 (p=0.002), and in parents from 3.34±0.79 to 3.66±0.79 (p=0.004). Consequently, the overall TAPS SUM score increased in children from 3.46±0.66 to 3.76±0.66 (p<0.001), and in parents from 3.27±0.72 to 3.67±0.70 (p<0.001) (Table 6, Figure 4).

**Figure 5:**
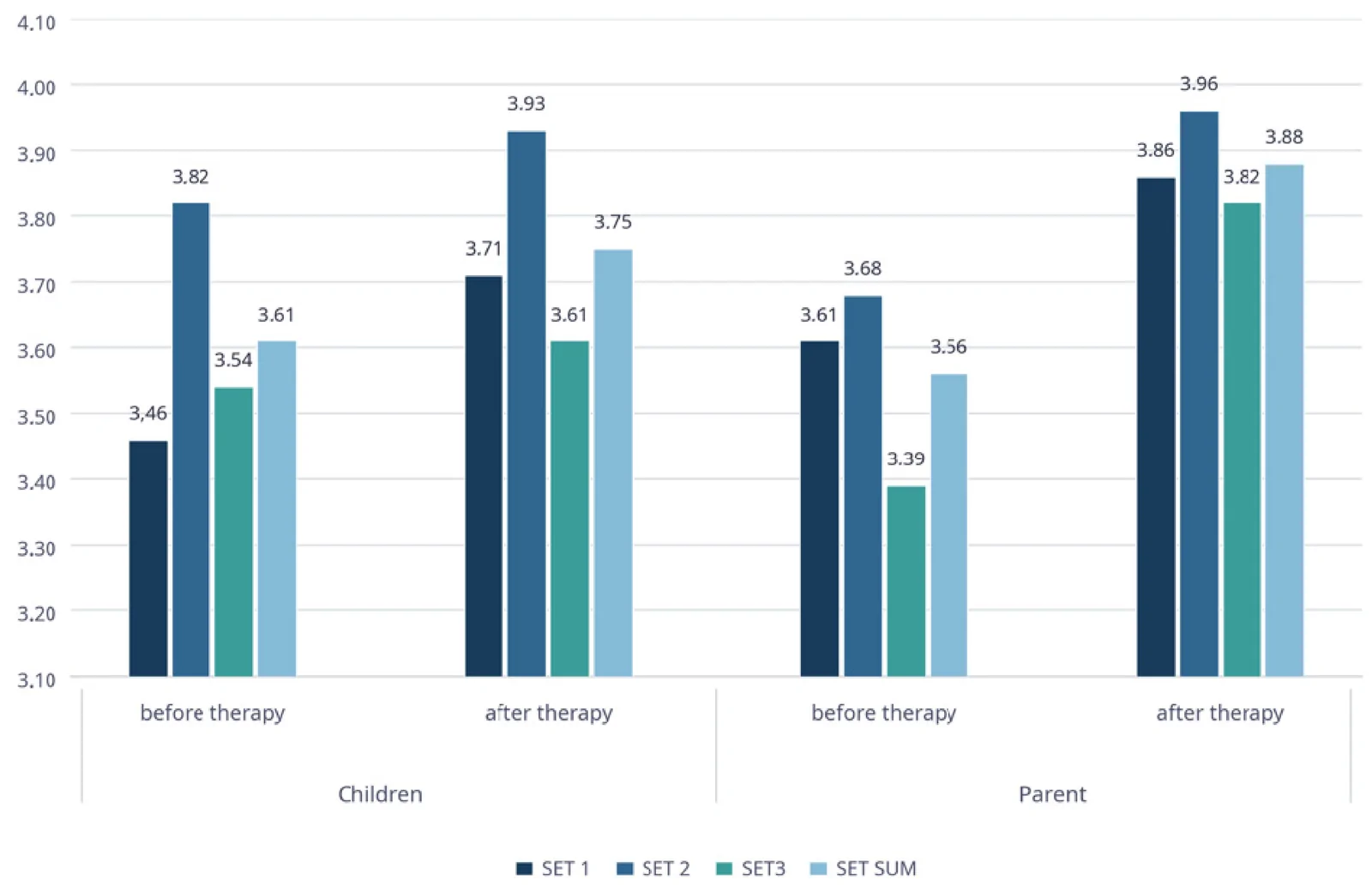
TAPS (SET 1, 2, 3) and TAPS SUM in non-braced children evaluated by children and parents before and after therapy.

In the non-braced group, the trajectory of changes was similar, though less pronounced. In children SET 1 scores increased from 3.46±0.88 to 3.71±0.76 (*p* = 0.132), SET 2 from 3.82±0.82 to 3.93±0.77 (p=0.495) and SET 3 from 3.54±0.79 to 3.61±0.63 (p=0.610) respectively; however, these differences were not statistically significant. Similarly, the TAPS SUM score in children increased from 3.61±0.67 to 3.75±0.59, but this change did not reach statistical significance (p=0.346). In parental evaluations, an increase was observed across all sets, reaching statistical significance for TAPS SUM, which increased from 3.56±0.66 to 3.88±0.62 (p=0.003) (Table 6, Figure 5).

Furthermore, no significant differences in TAPS SUM were found between children wearing braces and those not wearing braces, either before the camp (*p* = 0.367) or after its completion (*p* = 0.889). Regardless of whether the children wore a brace or not, perceived changes in body posture similarly.

Moreover, no significant differences were found between braced and non-braced children in the magnitude of change between the pre- and post-rehabilitation stay assessments (*p* = 0.947) (Table 7).

The comparison of changes in TAPS scores reported by children and parents was exploratory. Therefore, the results should be interpreted cautiously and regarded as hypothesis-generating rather than as evidence of differential treatment response.

## 4. Discussion

### 4.1. Key Results

Idiopathic scoliosis is not only a physical deformity of the spine but is also associated with asymmetries affecting the overall body shape. During adolescence, patients begin to pay greater attention to their appearance. Any asymmetries and deformities may therefore be noticed more readily and may affect the psychological well-being of the developing adolescent. Although conventional diagnostic methods, such as radiographs and clinical examinations, accurately determine the degree of spinal curvature and trunk deformity, they do not reflect the patient’s subjective perception of their own body. According to the SOSORT recommendations, scoliosis treatment should also aim to improve clinical appearance and the patient’s quality of life [16].

The analysis undertaken in the present study, which aimed to compare subjective assessments of body appearance made independently by patients and their parents, provides an important basis for considering the degree of agreement between these two groups in their perception of trunk deformity and the potential impact of any discrepancies on the rehabilitation process.

The available literature does not appear to include studies investigating the effect of scoliosis-specific physiotherapy on the assessment of a child’s appearance as perceived by both the children themselves and their parents before and after therapy. This makes it difficult for the authors to directly compare the present findings with those of previous studies.

O’Sanders et al. examined 182 patients and 133 parents. They completed the Walter Reed Visual Assessment Scale (WRVAS), which assessed the following aspects: body appearance from the back and front, appearance during forward bending with separate assessment of the rib prominence and lumbar prominence, trunk deviation from the midline, head position relative to the pelvis, and the level of the shoulders and scapulae. The results showed that patients with smaller spinal curves and those who had undergone surgery reported more favourable assessments than patients treated with a brace. Parents rated rib prominence and shoulder asymmetry less favourably than the children did. The remaining aspects of the deformity were assessed similarly by both groups [17]. It should be noted, however, that these assessments were not repeated after therapy, which limits the possibility of evaluating changes over time and makes direct comparison with the results obtained in the present study more difficult.

Our findings also confirmed that parents rated rib prominence less favourably than children before therapy. After the 5-day rehabilitation stay, both children and parents reassessed the child’s trunk appearance, and significantly higher scores were recorded in both groups, indicating a more favourable perception of the child’s appearance. Although parents continued to rate rib prominence slightly less favourably than children after therapy these differences were smaller. The assessments of the two groups became more similar, which represents a positive finding.

The lower pre-treatment ratings of rib prominence reported by parents may result from more frequent observation of the deformity at home or during medical appointments. Children may have greater difficulty observing their own deformity, particularly rib prominence. During the rehabilitation programme, however, the use of methods such as biofeedback may help them observe the deformity more clearly and become more aware of its severity.

In addition, both children and their parents actively participated in the therapeutic process during the rehabilitation programme and learned about the mechanisms underlying the deformity and the aims of treatment. Joint observation of treatment progress and education may contribute to more similar assessments of trunk appearance after therapy. Although these results indicate an improvement in the perception of appearance, the clinical significance of these changes has not yet been established. They suggest a temporal association rather than a causal effect of treatment.

Rigo et al. conducted two studies to determine whether discrepancies between children’s and parents’ assessments of trunk appearance were associated with quality of life in patients with idiopathic scoliosis. The first study included 71 patients and their parents. The mean age of the children was 17 ± 5.7 years, and the mean Cobb angle was 37 ± 15°. The second study included 107 girls and their parents. The mean age of the patients was 14.4 years, and the mean Cobb angle was 33.5° (range 10–75°). Both patients and parents independently completed the TAPS questionnaire. A TAPS discrepancy coefficient (TAPS-CD) was then calculated to reflect the difference between the child’s and the parent’s perception of the deformity. Quality of life was assessed using the SRS-22 questionnaire. The authors found that discrepancies in the perception of trunk deformity between children and their parents were associated with health-related quality of life. The greater the discrepancy between assessments, the poorer the patients’ body image and psychological well-being. They also emphasized that the assessment of trunk appearance should take into account both the patient’s and the parent’s perspectives. These studies demonstrate that agreement in the perception of deformity between a child and a parent has both clinical and psychosocial significance [18,19].

In our study, subjective assessments following the rehabilitation program were more favorable among both children and parents, although parents often rated the children more critically.

Chris Yin Wei Chan et al. examined 170 parent–child pairs and compared their perceptions of six physical characteristics in children with idiopathic scoliosis. The assessed characteristics included waist asymmetry, rib prominence, shoulder asymmetry, neck tilt, breast asymmetry, and anterior rib prominence. The overall Aggregate Physical Appearance score (Agg-PA), representing the overall importance attributed to physical appearance, was similar between patients and parents. Rib prominence and waist asymmetry were the greatest concerns for both children and parents [20].

Before the rehabilitation programme, children rated the appearance of their trunk during forward bending more favourably than their parents did. This may indicate that the children were less aware of the severity of the deformity or perceived it as less pronounced.

Misterska et al. conducted a prospective analysis involving 36 girls treated with a Chêneau brace, who were assessed at the beginning of therapy and after 6 and 12 months. The TAPS was used to assess the perception of trunk appearance. The authors demonstrated a significant improvement in TAPS scores during treatment, indicating a more favourable perception of body appearance as therapy progressed. Parents were not included in the assessment [21].

The study evaluated how the child and their parent perceived the trunk before and after the rehabilitation program, which may complement earlier observations.

Paolucci et al. conducted a study involving 32 patients with idiopathic scoliosis, including 16 patients treated with a brace and 16 non-braced patients. Perception of trunk appearance was assessed using the TAPS.

No statistically significant differences in TAPS scores were found between the braced and non-braced groups, which may indicate that brace treatment did not significantly affect the subjective perception of trunk appearance [22]. In our study involving 90 children (62 treated with a brace and 28 not treated with a brace), no statistically significant differences in body posture assessment were found between the two groups either.

A potential limitation of this study is selection bias. Participants were not randomly assigned to the braced and non-braced groups; brace use was determined by clinical indications and individual treatment decisions.

Therefore, the groups may have differed in unmeasured baseline characteristics, such as curve severity, skeletal maturity, treatment history, or adherence, which could have influenced the outcomes. The results may have been influenced by participant expectation bias related to participation and parents in rehabilitation and the subjective nature of the TAPS assessment. Consequently, comparisons between braced and non-braced participants should be interpreted with caution. It should be emphasized that subjective perception of trunk appearance and objective clinical postural parameters may reflect different aspects of functioning in patients with scoliosis and may not necessarily change in parallel, particularly after a short-term therapeutic intervention. The present study focused on changes in trunk appearance perception reported by children and their parents, as well as on the agreement between these assessments. An analysis of the relationship between changes in ATR and other clinical parameters and changes in TAPS scores was beyond the pre-specified objectives of this study. This issue warrants a separate, more comprehensive analysis in future research.

There is a lack of prospective studies that simultaneously monitor changes in the perception of trunk appearance in both children and parents from before the initiation of therapy through subsequent stages of conservative treatment until its completion.

The findings of the present study indicate that the assessment of trunk appearance is an important component of comprehensive treatment for patients with idiopathic scoliosis. The results suggest that joint participation by the child and parent in the therapeutic process, together with education about scoliosis, may contribute to greater agreement in assessing trunk appearance. The TAPS questionnaire may be a valuable tool that complements clinical assessment by accounting for individual perception of deformity.

### 4.2. Study Limitation

This study has limitations: retrospective design, short observation period, lack of control group, selection bias, expectation effect, lack of analysis of the relationship between changes in clinical parameters, such as ATR and changes in TAPS scores. The relationship between changes in clinical parameters, including ATR, and changes in TAPS scores was not analysed.

### 4.3. Clinical Implications and Future Perspectives

Children with scoliosis may experience substantial disturbances in body image, with several consequences. Objective measurements, such as the Cobb angle or angle of trunk rotation, often do not align with the patient’ s subjective assessment, and this discrepancy may be clinically relevant. The TAPS is a brief scale for assessing trunk appearance that allows clinicians to quickly determine how patients and their parents perceive the deformity. It is a useful supplementary assessment tool that may be applied in everyday clinical practice [23]. Completion of the questionnaire by both the child and the parent is important in the diagnostic and therapeutic process, as it may help identify a lack of awareness of the deformity, for example, when the child perceives the deformity as mild while the parent considers it severe. Such a discrepancy may indicate reduced awareness and insufficient education and, consequently, lower motivation to engage in treatment [14,18,19]. Cognitive concerns related to functioning may also be observed, including fears about losing physical attractiveness and body image disturbances [24]. A child may perceive the deformity as severe while the parent considers it mild. This may indicate reduced self- esteem and the need for psychological consultation. Marked discrepancies between parental and child ratings may indicate the need for further exploration of psychosocial concerns in the child or family [25]. Parental anxiety may increase the child’ s stress. Agreement between the child’ s and the parent’ s assessments facilitates the establishment of shared treatment goals, whereas substantial discrepancies may increase the risk of poor cooperation. Subjective assessment of trunk appearance should be regarded as an important component of comprehensive conservative treatment for idiopathic scoliosis [26]. Simultaneous TAPS assessment by both the patient and the parent may help identify differences in body image perception, awareness of the deformity, and psychosocial needs that radiological examinations alone cannot capture. Due to the lack of an established threshold for a minimally clinically important difference, the clinical significance of this change cannot be definitively determined. It is possible that a change in the score over a short period could serve a motivational purpose, reinforcing the engagement of children and their parents and encouraging them to continue treatment.

## 5. Conclusions

1. After a 5-day rehabilitation stay based on the FITS Method, both children with idiopathic scoliosis and their parents reported more favourable TAPS scores than before the programme.
2. Parents noticed greater improvement over time than children, but this result should be interpreted with caution and considered as a guide for further research.
3. After the rehabilitation stay, children treated with a brace and those who were not undergoing brace treatment reported similar perceptions of their trunk appearance.
4. Prospective studies with longer follow-up periods are needed to assess the durability of changes in perceived trunk appearance and to determine the long-term effects of scoliosis-specific physiotherapy on body image in patients with idiopathic scoliosis.

## Figures and Tables

**Table 1:** Descriptive statistics of the study group (*n* = 90).

Parameters	Number	Cobb Angle Average (SD)
Single-curve thoracic scoliosis	5	29.6 ± 16.5
Single-curve lumbar scoliosis	14	27.8 ± 7.1
Double-curve scoliosis:	71	
Thoracic Cobb		32.5 ± 12.9
Lumbar Cobb		32.2 ± 13.0
Risser		2.2 ± 1.9
Angle of Trunk Rotation (ATR) in Th		7.7 ± 4.4
Angle of Trunk Rotation (ATR) in L		4.7 ± 3.9
Brace	62	

**Table 2:** Results of the TAPS before therapy.

TAPS:	Average		
	Children	Parents	*p*
SET 1 (back)	3.37 ± 0.9	3.26 ± 0.9	*p* = 0.278
SET 2 (hump)	3.71 ± 0.7	3.48 ± 0.8	*p* = 0.022
SET 3 (front)	3.44 ± 0.8	3.36 ± 0.8	*p* = 0.347
SET SUM	3.51 ± 0.7	3.36 ± 0.6	*p* = 0.094

Test Wilcoxona.

**Table 3:** Clinical characteristics of the non-brace and brace groups.

Parameter	Non-Braced Group (*n* = 28)	Braced Group (*n* = 62)
Age	13.82 ± 2.48	13.50 ± 1.86
Risser	2.11 ± 1.85	2.28 ± 1.98
Curve pattern		
Double-curve	18 (64.3%)	53 (85.5%)
Single thoracic (TH)	2 (7.1%)	3 (4.8%)
Single thoracolumbar/lumbar (TH/L)	8 (28.6%)	6 (9.7%)
Cobb angle [°]		
Thoracic (TH)	25.95 ± 13.94 (*n* = 20)	34.55 ± 11.99 (*n* = 56)
Thoracolumbar/Lumbar (TH/L)	30.88 ± 14.64 (*n* = 26)	31.76 ± 11.36 (*n* = 59)
ATR [°]		
Thoracic (TH)	6.75 ± 4.50	8.34 ± 4.50
Lumbar (L)	6.18 ± 4.64	4.89 ± 3.21

**Table 4:** TAPS scores (SET 1, SET 2, SET 3 + TAPS SUM) reported by children and parents before and after therapy.

TAPS		SET SUM	U	U	SET 1	U	U	SET 2	U	U	SET3	U	U
			−95%	+95%		−95%	+95%		−95%	+95%		−95%	+95%
Children	before therapy	3.51 ± 0.66	3.37	3.65	3.37 ± 0.89	3.18	3.55	3.71 ± 0.71	3.56	3.86	3.44 ± 0.81	3.28	3.61
	after therapy	3.76 ± 0.64	3.62	3.89	3.71 ± 0.77	3.55	3.87	3.88 ± 0.73	3.72	4.03	3.68 ± 0.75	3.52	3.83
	*p* before-after	*p* < 0.001			*p* < 0.001			*p* = 0.041			*p* = 0.005		
Parent	before therapy	3.36 ± 0.71	3.21	3.51	3.26 ± 0.92	3.06	3.45	3.48 ± 0.81	3.31	3.65	3.36 ± 0.80	3.19	3.52
	after therapy	3.73 ± 0.68	3.59	3.88	3.67 ± 0.79	3.50	3.83	3.82 ± 0.76	3.66	3.98	3.71 ± 0.80	3.54	3.88
	*p* before-after	*p* < 0.001			*p* < 0.001			*p* = 0.001			*p* < 0.001		

Test Wilcoxona.

**Table 5:** Comparison of children’s and parents’ ratings for SET SUM.

Group	N	Mean	95% CI	SD
Children—before therapy	90	3.51	3.37–3.65	0.66
Children—after therapy	90	3.76	3.62–3.89	0.64
Parents—before therapy	90	3.36	3.21–3.51	0.71
Parents—after therapy	90	3.73	3.59–3.88	0.68
Children—difference before and after	90	0.25	0.13–0.36	0.55
Parents—difference before and after	90	0.37	0.25–0.49	0.59
Between-group comparison—before therapy			Z = 1.67; *p* = 0.094	
Between-group comparison—after therapy			Z = 0.43; *p* = 0.669	
Between-group comparison—difference before and after			Z = 1.99 *p* = 0.046	

Test Wilcoxona.

**Table 6:** TAPS scores (SET 1, SET 2, SET 3, and TAPS SUM) in braced and non-braced children, as assessed by children and parents before and after therapy.

TAPS			SET 1	SET 2	SET3	SET SUM
Children with Brace	Children	before therapy	3.32 ± 0.90	3.66 ± 0.65	3.40 ± 0.82	3.46 ± 0.66
		after therapy	3.71 ± 0.78	3.85 ± 0.72	3.71 ± 0.80	3.76 ± 0.66
		*p* before-after	*p* = 0.001	*p* = 0.039	*p* = 0.002	*p* < 0.001
	Parent	before therapy	3.10 ± 0.94	3.39 ± 0.80	3.34 ± 0.79	3.27 ± 0.72
		after therapy	3.58 ± 0.82	3.76 ± 0.76	3.66 ± 0.79	3.67 ± 0.70
		*p* before-after	*p* < 0.001	*p* = 0.003	*p* = 0.004	*p* < 0.001
Children Without Brace	Children	before therapy	3.46 ± 0.88	3.82 ± 0.82	3.54 ± 0.79	3.61 ± 0.67
		after therapy	3.71 ± 0.76	3.93 ± 0.77	3.61 ± 0.63	3.75 ± 0.59
		*p* before-after	*p* = 0.132	*p* = 0.495	*p* = 0.610	*p* = 0.346
	Parent	before therapy	3.61 ± 0.79	3.68 ± 0.82	3.39 ±0.83	3.56 ± 0.66
		after therapy	3.86 ± 0.71	3.96 ± 0.74	3.82 ± 0.82	3.88 ± 0.62
		*p* before-after	*p* = 0.062	*p* = 0.069	*p* = 0.005	*p* = 0.003

Test Wilcoxona.

**Table 7:** TAPS SUM scores in children with and without brace treatment.

Group	*n*	Mean	95% CI	SD
Children with brace—before therapy	62	3.46	3.29–3.63	0.66
Children with brace—after therapy	62	3.76	3.59–3.93	0.66
Children without brace—before therapy	28	3.61	3.35–3.87	0.67
Children without brace—after therapy	28	3.75	3.52–3.98	0.59
Children with brace—difference before and after	62	0.30	0.17–0.43	0.51
Children without brace—difference before and after	28	0.14	0.09–0.38	0.61
Between-group comparison—before therapy			Z = −0.90; *p* = 0.367	
Between-group comparison—after therapy			Z = −0.14; *p* = 0.889	
Between-group comparison—difference before and after			Z = 0.93; *p* = 0.947	

95% CI, 95% confidence interval; SD, standard deviation; Z, test U Manna–Whitney’a; *p*, *p*-value.

## Data Availability

The data presented in this study are available on request from the corresponding author due to legal and ethical reasons.
